# Preserved Cardiac Function despite Marked Impairment of cAMP Generation

**DOI:** 10.1371/journal.pone.0072151

**Published:** 2013-09-16

**Authors:** Mei Hua Gao, Ngai Chin Lai, Tong Tang, Tracy Guo, Ruoying Tang, Byeong Jo Chun, Hong Wang, Nancy N. Dalton, Jorge Suarez, Wolfgang H. Dillmann, H. Kirk Hammond

**Affiliations:** VA San Diego Healthcare System and Department of Medicine, University of California San Diego, San Diego, California, United States of America; Temple University, United States of America

## Abstract

**Objectives:**

So many clinical trials of positive inotropes have failed, that it is now axiomatic that agents that increase cAMP are deleterious to the failing heart. An alternative strategy is to alter myocardial Ca^2+^ handling or myofilament response to Ca^2+^ using agents that do not affect cAMP. Although left ventricular (LV) function is tightly linked to adenylyl cyclase (AC) activity, the beneficial effects of AC may be independent of cAMP and instead stem from effects on Ca^2+^ handling. Here we ask whether an AC mutant molecule that *reduces* LV cAMP production would have favorable effects on LV function through its effects on Ca^2+^ handling alone.

**Methods and Results:**

We generated transgenic mice with cardiac-directed expression of an AC6 mutant (AC6mut). Cardiac myocytes showed impaired cAMP production in response to isoproterenol (74% reduction; p<0.001), but LV size and function were normal. Isolated hearts showed preserved LV function in response to isoproterenol stimulation. AC6mut expression was associated with increased sarcoplasmic reticulum Ca^2+^ uptake and the EC_50_ for SERCA2a activation was reduced. Cardiac myocytes isolated from AC6mut mice showed increased amplitude of Ca^2+^ transients in response to isoproterenol (p = 0.0001). AC6mut expression also was associated with increased expression of LV S100A1 (p = 0.03) and reduced expression of phospholamban protein (p = 0.01).

**Conclusion:**

LV AC mutant expression is associated with normal cardiac function despite impaired cAMP generation. The mechanism appears to be through effects on Ca^2+^ handling — effects that occur despite diminished cAMP.

## Introduction

So many clinical trials of positive inotropes have failed, that it is now axiomatic that agents that increase cAMP are deleterious to the failing heart. An alternative strategy is to alter myocardial Ca^2+^ handling or myofilament response to Ca^2+^ using agents that do not affect cAMP [1]. Although it is a hallmark of classical physiology that left ventricular (LV) function is dictated by adenylyl cyclase (AC) activity, the effects of AC on the heart, recent studies indicate, may be more related to effects on Ca^2+^ handling than cAMP production *per se* [2],[3],[4],[5]. This speculation requires further examination both because of its physiological impact and its potential therapeutic implications. After all, despite steady improvement in drug and device therapy for CHF—including LV assist devices—the prognosis for CHF patients remains dismal and major new classes of pharmacological agents have not been introduced for many years. In the present study we ask whether an AC mutant molecule that *reduces* LV cAMP generating capacity might have favorable effects on LV function through its direct effects on Ca^2+^ handling alone.

Data from previous studies indicate that increased cardiac AC type 6 (AC6), a dominant AC isoform expressed in mammalian cardiac myocytes [6], has protean beneficial effects on the failing left ventricle (LV) [7],[8],[9],[10],[11],[12]. These unexpected beneficial effects must be reconciled with the dire consequences on the heart of βAR stimulation and elevations in intracellular cAMP [13],[14],[15],[16],[17],[18]. Indeed, the apparent benefits of AC6 expression in the failing heart is paradoxical. Using pharmacological inhibitors, data from previous studies suggest that some of the beneficial effects of increased cardiac AC6 expression do not depend upon increased cAMP generation [2],[3]. Because of the inherent limitations of studies using pharmacological inhibition in cultured cardiac myocytes, we generated a catalytically inactive AC6 mutant (AC6mut) molecule by substitution of Ala for Asp at position 426 in the catalytic core, a change predicted to alter Mg^2+^ binding but not influence G-protein dynamics [4]. This AC6mut molecule, when studied *in vitro*, markedly impairs cAMP generation, but retains the cellular distribution pattern associated with AC6 [4]. Such *in vitro* studies fall far short of establishing how such a molecule might influence cardiac function *in vivo*.

We therefore generated transgenic murine lines with cardiac-directed expression of AC6mut. Our hope was that such lines would provide additional insight vis-à-vis differentiation of cAMP vs Ca^2+^ handling effects on the function of the intact normal heart. Furthermore, such studies might indicate whether AC6mut provides inotropic stimulation free from the potentially deleterious effects of increased cAMP. Our hypothesis was that LV function, despite marked diminution in cAMP generating capacity, would remain normal, through beneficial counterbalancing effects conferred by AC6 on Ca^2+^ handling.

## Methods

### Generation of AC6mut Transgenic Mice (Fig. 1A)

The use of animals was in accordance with Association for Assessment and Accreditation of Laboratory Animal Care guidelines and was approved by the Institutional Animal Care and Use Committee of VA San Diego Healthcare System. To generate mice with cardiac-directed expression of AC6mut, the murine AC6mut cDNA [4] with an AU1 tag at the C-terminus, was subcloned between the α-myosin heavy chain promoter and SV40 polyA. A 9.2-kb fragment containing the expression cassette was used for pronuclear injection, carried out in the transgenic mouse facility at University of California, San Diego (inbred C57BL/6). Founder mice were identified by polymerase chain reaction (PCR) of genomic DNA prepared from tail tips. The AC6mut gene was detected using a primer homologous to the α-MHC promoter (forward: 5′ CACATAGAAGCCTAGCCCACACC); the reverse primer was for the AC6 region (5′ CAGGAGGCCACTAAACCATGAC). AC6mut mRNA was detected using the following primers: (forward: 5′ TGGGCCTCTCTACTCTGCAT; reverse: 5′ TGGATGTAACCTCGGGTCTC) enabling quantification of fold increase of AC6mut mRNA over endogenous AC6 mRNA. Endogenous AC6 mRNA was detected using primers homologous to its 3′-untranslated region (forward: 5′ GGCATTGAGTGGGACTTTGT; reverse: 5′ TCTGCATCCAAACAAACGAA). This 3′ untranslated region was not present in the AC6mut cDNA, enabling quantification of endogenous AC6. Founder animals were crossbred with wild-type mice of the same strain, and selected animals were used for analysis of cardiac transgene expression. We documented variable transgene expression in independent lines and selected a line with a 17-fold increase in AC6mut protein expression (vs endogenous AC6) for our studies. LV expression levels of AC types 2–9 were determined using quantitative RT-PCR as previously described [5].

**Figure 1:**
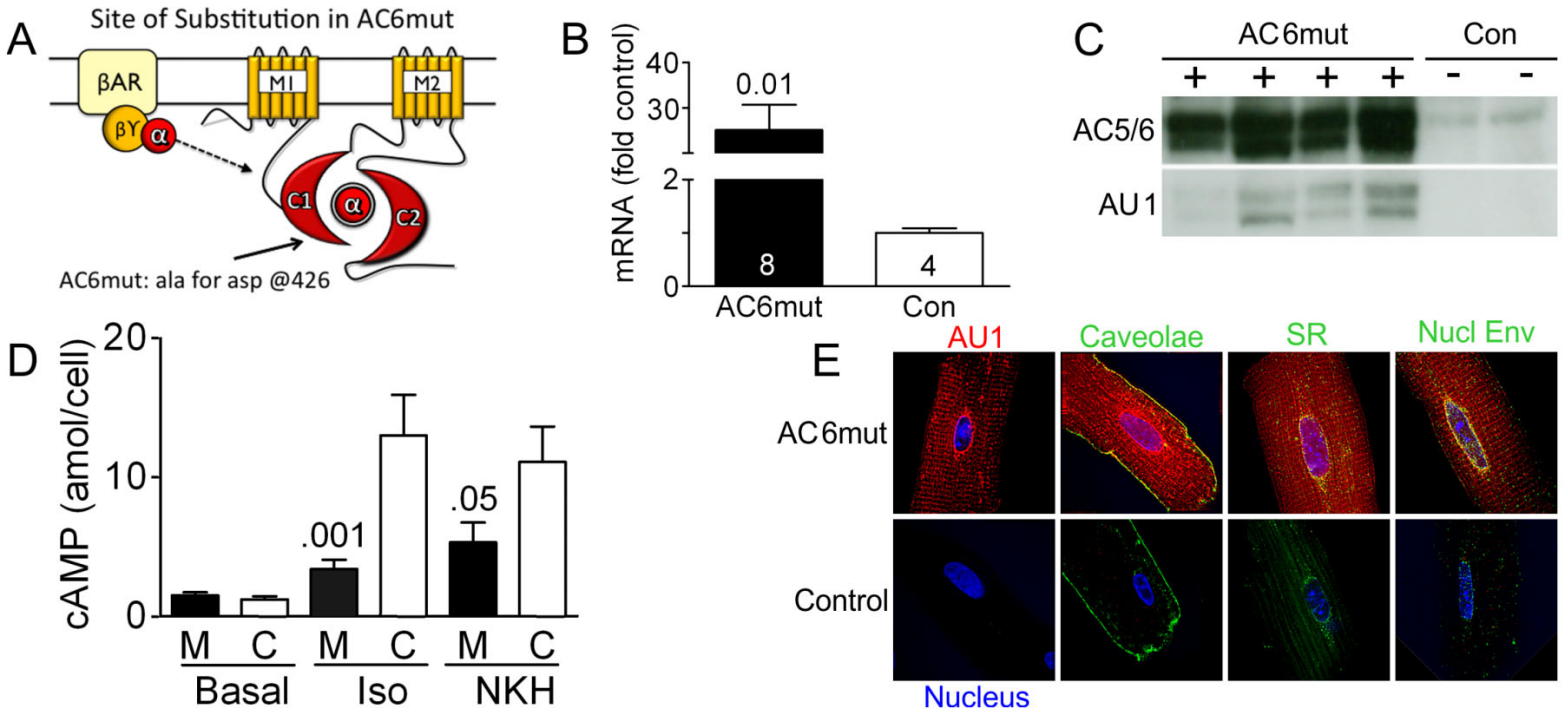
AC6mut Design, Expression, Activity and Cellular Distribution. **A**. The diagram depicts the site of substitution of alanine (ala) for aspartic acid (asp) at position 426 in the C1 domain (intracellular loop) in the construction of AC6mut. The substitution inhibits Mg^2+^ binding and alters the efficiency of Gsα-mediated activation of the catalytic core, which impairs the enzymatic activity of AC6, resulting in reduced cAMP production. M1 and M2, transmembrane domains of AC6; C1 and C2, cytoplasmic domains of AC6, which form the catalytic core; βAR, β-adrenergic receptor; βΥ and α, components of the guanosine 5′-triphosphate (GTP)-binding protein, Gs. **B**. AC6mut mRNA expression was assessed by qRT-PCR using primers common to endogenous AC6 and transgene AC6mut. Primers for detecting GAPDH mRNA were used for internal control of the qRT-PCR reaction. AC6mut mRNA was increased 62-fold vs endogenous AC6. Animal number in bars +SE; Student's t-test, unpaired, 2 tails. **C**. AC6mut protein was detected in immunoblotting using anti-AC5/6 antibody and confirmed using anti-AU1 tag antibody. AC6mut protein was increased 17-fold vs endogenous AC6. **D**. Cyclic AMP production in isolated cardiac myocytes from AC6mut and control mice, before (Basal) and after stimulation with isoproterenol (Iso; 10 µM, 10 min) or NKH477 (NKH; 10 µM, 10 min), using cAMP Enzymeimmunoassay. Cardiac myocytes from AC6mut mice (M vs C, control) showed impaired cAMP production in response to Iso and NKH477, a forskolin analog. Bars denote mean +SE; p values from 1-way ANOVA followed by Bonferroni post test (n = 6, each group). **E**. Double immunofluorescence staining of AC6mut protein in cardiac myocytes isolated from AC6mut vs control mice using anti-AU1 antibody (red); anti-caveolin 3 (Cav-3) antibody (green, for caveolae); anti-protein disulphide-isomerase (PDI) antibody (green, for sarcoplasmic reticulum); and anti-lamin A antibody (green, for nuclear envelope). Nucleus is blue. AC6mut transgene was detected in caveolae, SR, and nuclear envelope.

### Echocardiography

Anesthesia was induced with 5% isoflurane (at a flow rate of 1 L/min oxygen) and maintained with 1% isoflurane in oxygen. Images were obtained using a 16 L MHz linear probe and Sonos 5500® Echocardiograph system (Philips Medical Systems, Bothell, WA), as previously reported [7]. Data were acquired and analyzed without knowledge of group identity.

### Isolated Perfused Hearts: LV Contractile Function

Cardiac function was assessed in isolated perfused hearts to assess LV contractile function in a manner unaffected by reflex activation or anesthesia, as previously reported [7]. An intraventricular balloon catheter was deployed to measure isovolumic LV pressure (LV end-diastolic pressure 10 mmHg; 1.7 mM ionized Ca^2+^). Isoproterenol was delivered in bolus doses (from 0.1 nM to 300 nM) at five-minute intervals as LV pressure was recorded. Subsequently, the first derivative of the LV pressure (LV dP/dt) was derived and used as a surrogate of LV contractile function. Data were acquired and analyzed without knowledge of group identity.

### Calcium Uptake

Initial rate of ATP-dependent sarcoplasmic reticulum (SR) Ca^2+^ uptake in LV homogenates was measured by the modified Millipore filtration technique as described previously [11].

### Calcium Transient

Cytosolic calcium transients were measured using Indo-1, as described previously [19]. Cardiac myocytes were plated onto laminin-coated glass cover slips and loaded with indo-1/AM (3 µM, Calbiochem, La Jolla CA) and dispersing agent, pluronic F-127 (0.02 mg/ml, Calbiochem, La Jolla CA) for 30 min. Following dye loading, cover slips were mounted in a superfusion chamber, rinsed to remove excess indo-1/AM, and mounted on a Nikon Diaphot epifluorescence microscope equipped with a 40× objective interfaced to a Photon Technologies photometry system (Birmingham NJ) with the excitation wavelength set to 365 nm via a monochromator. Fluorescence emission was split and directed to two photomultiplier tubes through 20-nm band-pass filters centered at 405 and 485 nm, respectively. The ratio F405/F485 represents a measure for [Ca^2+^]i. During these measurements, cardiac myocytes were superfused with 25 mM HEPES (pH 7.3) containing 2 mM CaCl_2_. Myocytes were field-stimulated at 0.3 Hz. Isoproterenol-stimulated Ca^2+^ transient was determined by adding isoproterenol (10 µM) to the buffer. Calcium transients were recorded from at least 20 cells per heart and for at least 3 hearts per group. Diastolic and systolic intracellular Ca^2+^ levels were obtained from the basal and maximal F405/F485 ratio per cycle, respectively.

### Cardiac Myocyte Isolation

Cardiac myocyte isolation was performed as previously described [4].

### Cyclic AMP Measurement

Isolated cardiac myocytes were stimulated with isoproterenol (10 µM, 10 min) or the water-soluble forskolin analog NKH477 (10 µM, 10 min), and then lysed (2.5% dodecyltrimethylammonium bromide, 0.05 M sodium acetate, pH 5.8, and 0.02% bovine serum albumin). Cyclic AMP was measured using the cAMP Biotrak Enzymeimmunoassay System (GE Healthcare) as previously reported [4].

### PKA Activity Assay

Isolated cardiac myocytes were stimulated with isoproterenol (10 µM, 10 min) or NKH477 (10 µM, 10 min). Cardiac myocytes were homogenized in buffer A: 20 mM Tris-HCl (pH 7.4), 0.5 mM EGTA, 0.5 mM EDTA, and protease inhibitor cocktail from Invitrogen) and centrifuged (14,000×*g*, 5 min, 4°C). The supernatant was incubated with PKA biotinylated peptide substrate (SignaTECT® cAMP-Dependent Protein Kinase Assay System, Promega, Madison WI) in the presence of [γ-^32^P]ATP. The ^32^P-labeled, biotinylated substrate was recovered with a streptavidin matrix, and the specific activity of PKA determined.

### Isoproterenol-Stimulated Phosphorylation of Ryanodine Receptor-2, PLB, and Troponin I in Cardiac Myocytes

**Figure 2:**
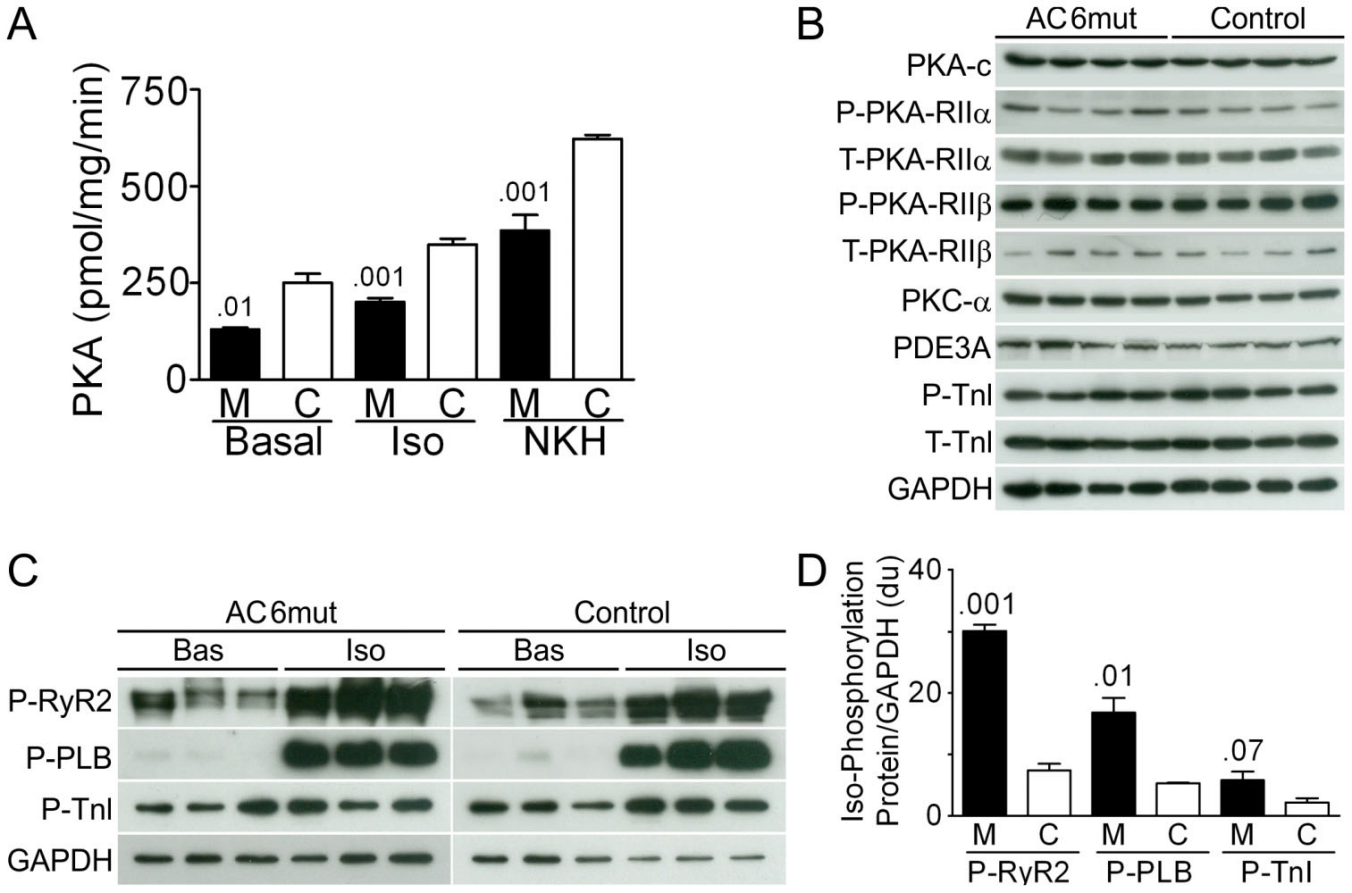
Activities and Expression of PKA, PKS and PDE. **A**. PKA activity in isolated cardiac myocytes without stimulation (Basal) or stimulated with isoproterenol (Iso; 10 µM, 10 min) or NKH477 (NKH; 10 µM, 10 min). AC6mut expression reduced basal PKA activity (p = 0.01) and both Iso (p = 0.001) and NKH (p = 0.001) activities were reduced as well (n = 3, each group). **B**. The expression of key signaling proteins and their phosphorylation are shown in immunoblots using left ventricular homogenates from AC6mut and control mice. No group differences were observed. Shown are PKA catalytic unit, phospho (P) and Total (T) PKA regulatory subunits II-α and II-β, PKCα, phosphodiesterase type 3A (PDE3A), phospho-troponin I (P22/23-TnI), and total TnI. **C**. Phosphorylation of RyR2, PLB and TnI before and after isoproterenol stimulation was assessed in cardiac myocytes isolated from each group. Basal phosphorylation of RyR2, PLB and TnI showed no group differences. Isoproterenol stimulation was associated with increased phosporylation of RyR2, PLB, and TnI in both groups, but was more extensive in cardiac mycoytes from AC6mut mice. **D**. The data from **Fig. 2C** indicating that isoproterenol stimulation was associated with increased phosporylation of RyR2, PLB, and TnI in cardiac mycoytes from AC6mut mice are shown in graphic format, normalized for loading (GAPDH). The increase in TnI phosphorylation was not statistically significant (p = 0.07).

To determine dynamic phosphorylation of key Ca^2+^ regulating proteins, we conducted studies of basal and isoproterenol-stimulated phosphorylation of RyR2, PLB and TnI in cultured cardiac myocytes isolated from each group (**Fig. 2C**). Cultured cardiac myocytes (100,000 cells per well) were used in these studies and immunoblotting performed before and after incubation with isoproterenol (10 µM, 10 min). Cells were lysed in lysis buffer: 20 mM Tris-HCl (pH 7.5), 150 mM NaCl, 1 mM Na_2_EDTA, 1 mM EGTA, 1% Triton, 2.5 mM sodium pyrophosphate, 1 mM β-glycerophosphate, 1 mM Na_3_VO_4_, 1 µg/ml leupeptin). Protein concentration was measured using the Bradford method. Immunoblots were normalized to GAPDH and compared (**Fig. 2D**).

### PDE Activity Assay

Phosphodiesterase activity was assayed using the Cyclic Nucleotide Phosphodiesterase Assay Kit (Enzo). LV tissues were homogenized in buffer containing 10 mM Tris-HCl (pH 7.4), 1 mM PMSF, 10 mM activated orthovanadate, 1× protease inhibitor cocktail (Life Sciences) and centrifuged at 10,000 rpm (10 min) in a microfuge. Tissue homogenates were desalted by gel filtration using Desalting Column Resin (Enzo). Twenty µg of protein (Bradford) was added to each well and PDE activity measured.

### Immunofluorescence

Isolated cardiac myocytes were attached to laminin coated 2-well chamber slides for 1 hr, washed, fixed (10% formalin, 15 min, 23°C), blocked with normal goat serum (1 hr) and incubated (4°C, overnight) with: anti-AU1 antibody (Fitzgerald, 1∶300; for detecting AC6mut transgene protein); anti-Cav3 antibody (BD Pharmagen, 1∶100; for detecting caveolae); anti-PDI antibody (Invitrogen, 1∶1000; for detecting SR); anti-lamin A (Abcam, 1∶200; for detecting nuclear envelope); anti-CREM-1 antibody (Santa Cruz, 1∶50); or anti-phospho-CREB antibody (Upstate, 1∶100). Cardiac myocytes were washed with PBS and then incubated with secondary antibodies (Alexia Fluo 488 or 594 conjugated, 1∶1000 dilution) for 1 hr. To identify the nucleus, cells were incubated with Hoechst dye (1∶1000 dilution, 20 min). Cardiac myocytes then were imaged as previously described [2].

### Detection of mRNA and Immunoblotting

Quantitative reverse transcription polymerase chain reaction (RT-qPCR) was used to quantify mRNA and immunoblotting was used to quantify protein content [4]. The primers for RyR2 included (forward: 5′AACCTACCAGGCTGTGGATG); and (reverse: 5′ GACTCGATGGGCAAGTCAAT).

We used the anti-AC5/6 antibody to identify endogenous AC6 and AC6mut (Santa Cruz, 1∶200 dilution). The epitope for the AC5/6 antibody is at the C-terminus of AC6 and AC6mut (sequence: KGYQLECRGVVKVKGKGEMTTYFLNGGPSS; protein accession #O43306 and #Q01234). We used AU1 antibody (Fitzgerald, 1∶2,000) to detect AC6mut protein. Additional antibodies used included: calreticulin (ABR Affinity, 1∶1,000); calsequestrin (Novus Biologicals, 1∶1,000); GAPDH (Fitzgerald, 1∶20,000); PDE3A (Santa Cruz, 1∶500); PKA catalytic subunit (BD Transduction, 1∶1,000); p-PKA catalytic subunit (Cell Signaling, 1∶1,000); PKA-RIIα and PKA-RIIβ (BD Transduction, 1∶1,000); phospho-PKA-RIIα (S96) and phospho-PKA-RIIβ (S114) (Santa Cruz, 1∶200); PKCα catalytic subunit (Santa Cruz, 1∶200); PLB (Affinity Bioreagents, 1∶5,000); phospho S16-PLB (Badrilla, 1∶3,000 dilution); phospho-RyR2 (S2808) (Abcam, 1∶1,000); S100A1 (Epiyomics, 1∶1,000); SERCA2a (Enzo, 1∶1,000); troponin I and phospho-TnI (S22/23) (Cell Signaling, 1∶1,000 each).

### Statistical Analysis

Data represent mean ± SE; group differences were tested for statistical significance using either ANOVA, followed by Bonferroni *t*-testing, or, when appropriate, Student's *t* test (unpaired, 2-tailed). The null hypothesis was rejected when p<0.05.

## Results

### AC6mut Transgenic Mice

AC6mut mice were physically indistinguishable from their transgene negative siblings. Necropsy of adult mice showed that body weight, tibial length, LV weight, and lung weight showed no group differences. (**Table 1**).

**Table 1:** Body, LV, and Lung Weight.

	AC6mut (23)	TG- Control (16)	p
Body (g)	25.5±0.7	25.0±1.2	0.7
LV (mg)	91±2.7	89±3.4	0.6
Tibial Length (mm)	17±0.1	16.7±0.2	0.3
LV/Body (mg/g)	3.6±0.1	3.6±0.1	0.9
LV/TL (mg/mm)	5.4±0.1	5.3±0.2	0.7
Lung (mg)	150±4.9	149±6.7	0.9
Lung/Body (mg/g)	6.0±0.2	6.0±0.2	0.9

LV, left ventricle; TL, tibial length. Values represent mean ± SE; Student's *t* test (unpaired, 2-tailed).

#### LV Expression of AC6mut

AC6mut mRNA was increased 62-fold and protein was increased 17-fold over the levels of endogenous AC6, which were detected using primers and antibody to the common regions on both endogenous AC6 and transgene AC6mut in RT-PCR and immunoblotting (**Figs. 1B and 1C**).

#### LV Expression of Endogenous AC Types

The mRNA of endogenous AC types 2–9 showed no group differences (data not shown).

#### LV cAMP Production

LV samples from AC6mut mice showed reduced cAMP production when stimulated with isoproterenol (74% reduction; p<0.001) or NKH477, a water-soluble forskolin analog (52% reduction; p = 0.05) (**Fig. 1D**); basal cAMP production was unchanged. Thus, the transgenic line was suited to test the overall effect of reduced βAR-stimulated cAMP production in the presence of increased AC6mut expression on LV function.

#### PKA Activity and Expression

Cardiac myocytes isolated from AC6mut mice showed a 48% reduction in basal PKA activity (p = 0.01). In addition there were reductions in PKA activity stimulated by isoproterenol (38% reduction; p = 0.006); and NKH477 (38% reduction; p = 0.001) (**Fig. 2A** **, upper**). AC6mut expression did not alter LV expression of the PKA catalytic subunit (**Fig. 2B**) or expression or phosphorylation of PKA-RII-α and β (phospho-PKA-RIIα: AC6mut, 0.32±0.04 du; Con, 0.30±0.03 du, p = 0.7; phospho-PKA-RIIβ: AC6mut, 7.1±1.1 du; Con, 10.6±01.4 du; p = 0.09; **Fig. 2B**). PKC catalytic subunit expression also showed no group difference (PKCα: AC6mut, 0.8±0.1 du; Con, 0. 7±0.1 du, p = 0.4; **Fig. 2B**).

#### Isoproterenol-Stimulated Phosphorylation of Ryanodine Receptor-2, PLB and Troponin I in Cardiac Myocytes

Basal phosphorylation of RyR2, PLB and TnI showed no group differences (P-RyR2: AC6mut, 4.4±0.6 vs Con, 2.4±0.5 du, p = 0.06; P-PLB: AC6mut, 0.3±0.03 vs Con, 0.2±0.1 du, p = 0.8; P-TnI: AC6mut, 0.8±0.2 vs Con, 1.0±0.01 du, p = 0.24, **Fig. 2C**). Isoproterenol stimulation was associated with increased phosporylation of RyR2, PLB, and TnI in both groups (vs unstimulated), but the extent of phosphoryaltion generally was greater in LV from AC6mut mice (P-RyR2: AC6mut, 30.0±1.1vs Con, 7.4±1.1 du, p = 0.001,; P-PLB: AC6mut, 16.8±2.4 vs Con, 5.3±0.1 du, p = 0.01; P-TnI: AC6mut, 5.8±1.4 vs Con, 2.2±0.7 du, p = 0.07; **Fig. 2C**). TnI protein expression was not different between groups (AC6mut, 0.9±0.1 vs Con, 0.7±0.2 du; p = 0.5; **Fig. 2B**. RyR2 mRNA expression showed no group difference (data not shown).

#### PDE Activity and PDE3A Expression

There was no group difference in PDE activity in LV samples (AC6mut: 1252±23 Units/mg, n = 7; Control: 1293±39 Units/mg, n = 6; p = 0.38). LV PDE3A protein expression showed no group difference (AC6mut: 0.3±0.1 vs Con, 0.4±0.1 du, p = 0.6. **Fig. 2B**).

#### Intracellular Distribution of AC6mut

AC6mut protein was identified in association with caveolae (mainly associated with plasma membrane), SR, and nuclear envelope (**Fig. 1E**).

### Echocardiography

Echocardiography showed that basal cardiac structure and function were unchanged by cardiac-directed expression of AC6mut. LV dimensions were not different between groups, and basal LV ejection fraction and the velocity of circumferential fiber shortening were similar (**Table 2**). Thus, despite marked diminution of LV cAMP generating capacity in AC6mut mice, LV structure and basal function were unaltered.

**Table 2:** Echocardiography (Basal).

	AC6mut (8)	TG- Control (12)	p
HR (bpm)	501±26	506±17	0.9
EDD (mm)	4.2±0.2	4.3±0.1	0.7
ESD (mm)	2.9±0.2	3.0±0.1	0.4
PW Thickness (mm)	0.6±0.1	0.6±0.1	0.5
Septal Thickness (mm)	0.6±0.1	0.6±0.1	0.4
EDV (µL)	76±7	78±4	0.8
ESV (µL)	25±4	27±2	0.6
EF (%)	69±3	65±2	0.2
CO (µL/min)	26±2	26±2	0.8
Vcf (circ/sec)	7.0±0.7	6.2±0.3	0.2

### LV Contractile Function in Response to Isoproterenol

**Figure 3:**
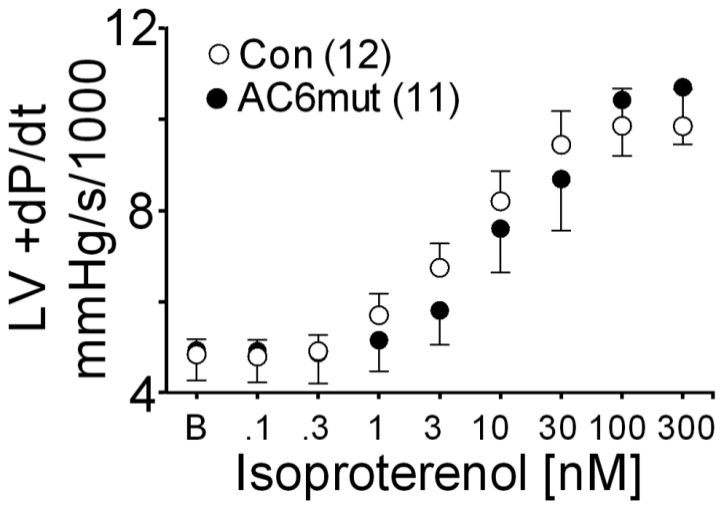
Left Ventricular Contractile Function. Isolated hearts from AC6mut TG mice (closed circle; n = 11) showed preserved LV dP/dt in response to isoproterenol stimulation through a wide range of isoproterenol doses. Data were acquired and analyzed without knowledge of group identity. Open circles, transgene negative control mice (n = 12). There was no group difference (2-way ANOVA). Data points denote mean ±SE.

To assess cardiac contractility in a manner independent of autonomic nervous influence, endogenous catecholamines, and anesthesia, LV pressure development was measured in isolated perfused hearts. Basal and isoproterenol-stimulated LV dP/dt showed no group differences (**Fig. 3**), despite marked diminution in LV cAMP generating capacity.

### Ca^2+^ Uptake and Ca^2+^ Related Proteins

**Figure 4:**
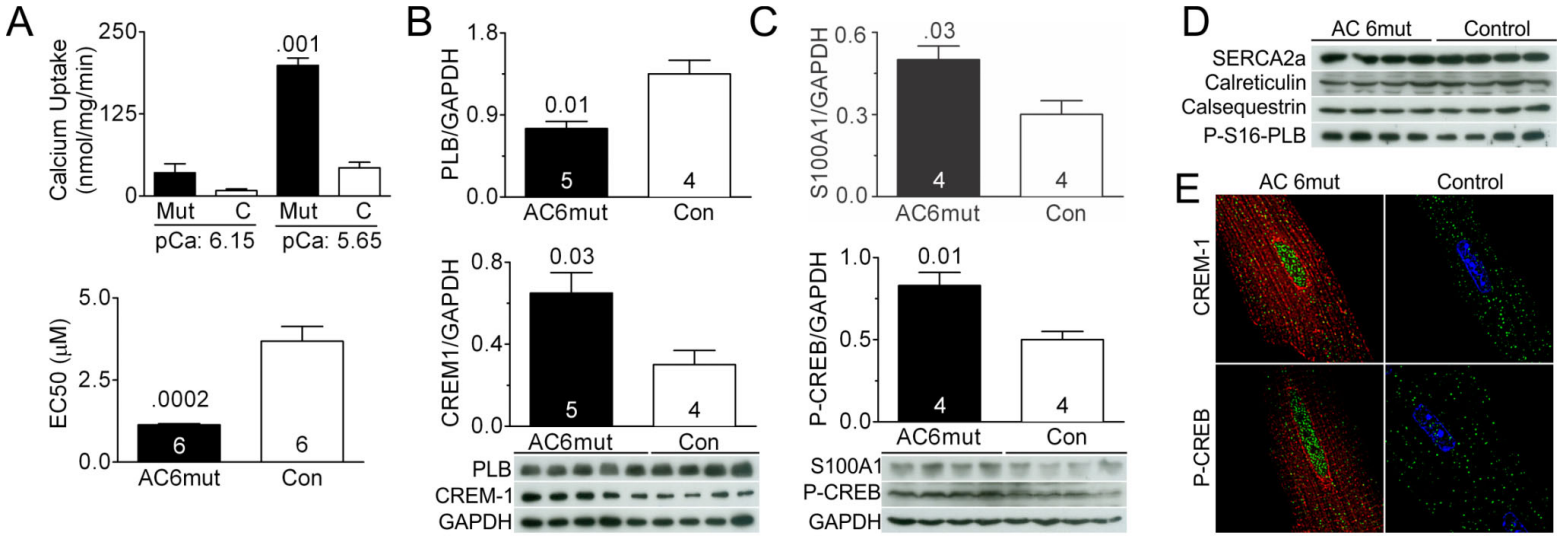
SR Ca^2+^ uptake, Ca^2+^ signaling proteins, and transcriptional factors. **A**. **Upper:** Ca^2+^ uptake activity in pooled LV samples from AC6mut and TG negative sibling control mice (n = 6, both groups). **Lower:** Expression of AC6mut decreased SERCA2a affinity for Ca^2+^. The effective concentration of Ca^2+^ for 50% maximal effect (EC_50_) was calculated from the initial ATP-dependent Ca^2+^ uptake rate at different free Ca^2+^ concentrations. **B**. **Upper:** AC6mut expression was associated with decreased LV phospholamban (PLB) expression. **Lower:** AC6mut expression was associated with increased LV CREM-1 protein expression. **C**. **Upper:** AC6mut expression was associated with increased LV S100A1 protein expression. **Lower:** AC6mut expression was associated with increased LV P133-CREB protein phosphorylation. Total CREB expression was similar in both groups. **D**. AC6mut expression did not affect LV expression of SERCA2a, calreticulin, calsequestrin or phospho-S16-PLB proteins. (n = 4, both groups). **E**. Double immunofluorescence staining of AC6mut protein in isolated cardiac myocytes from AC6mut and control mice using anti-AU1 antibody (red) and anti–CREM-1 antibody (green) or anti-AU1 and anti-phospho-CREB (S133, green). Nucleus was showing in blue. AC6mut expression increased nuclear localizations of CREM-1 and phospho-CREB. In graphs (A,B,C), bars denote mean +SE; numbers in bars indicate group size; members above bars indicate p values from Student's t-test (unpaired, 2 tailed).

ATP-dependent SR Ca^2+^ uptake rate in pooled LV homogenates from AC6mut and transgene-negative sibling control mice was determined. Increased AC6mut expression was associated with increased SR Ca^2+^ uptake (**Fig. 4A** **, upper panel**). In addition, an increased affinity of SERCA2a for Ca^2+^ was reflected in a reduced Ca^2+^ concentration required for a half maximal effect (EC50: AC6mut 1.1 µmol/L; Control 3.7 µmol/L, n = 6, **Fig. 4A** **, lower panel**).

Associated with these physiological changes in Ca^2+^ handling was altered LV expression of proteins that regulate SR Ca^2+^ uptake. For example, AC6mut expression was associated with a 43% reduction in LV PLB protein expression (p = 0.01), and a 73% increase in LV S100A1 protein content (p = 0.03) (**Figs. 4B and 4C**). The contents of LV SERCA2a, calreticulin, and calsequestrin were unchanged, and PLB phosphorylation at Ser16 was unchanged (**Fig. 4D**).

### Transcription Factors

AC6mut expression was associated with a 2-fold increase in LV expression of CREM-1 (p = 0.03, **Fig. 4B**) and a 1.7-fold increase in phosphorylation of CREB at Ser133 (p = 0.01, **Fig. 4C**); total CREB protein content was unaltered. To determine whether increased CREM-1 and phospho-CREB were present in the nuclei, immunofluorescence staining of isolated cardiac myocytes was performed using anti-CREM-1 and anti-phospho-CREB (S133) antibodies. We detected increased nuclear localization of CREM-1 and phospho-CREB in AC6mut mice (**Fig. 4E**).

### Calcium Transients

**Figure 5:**
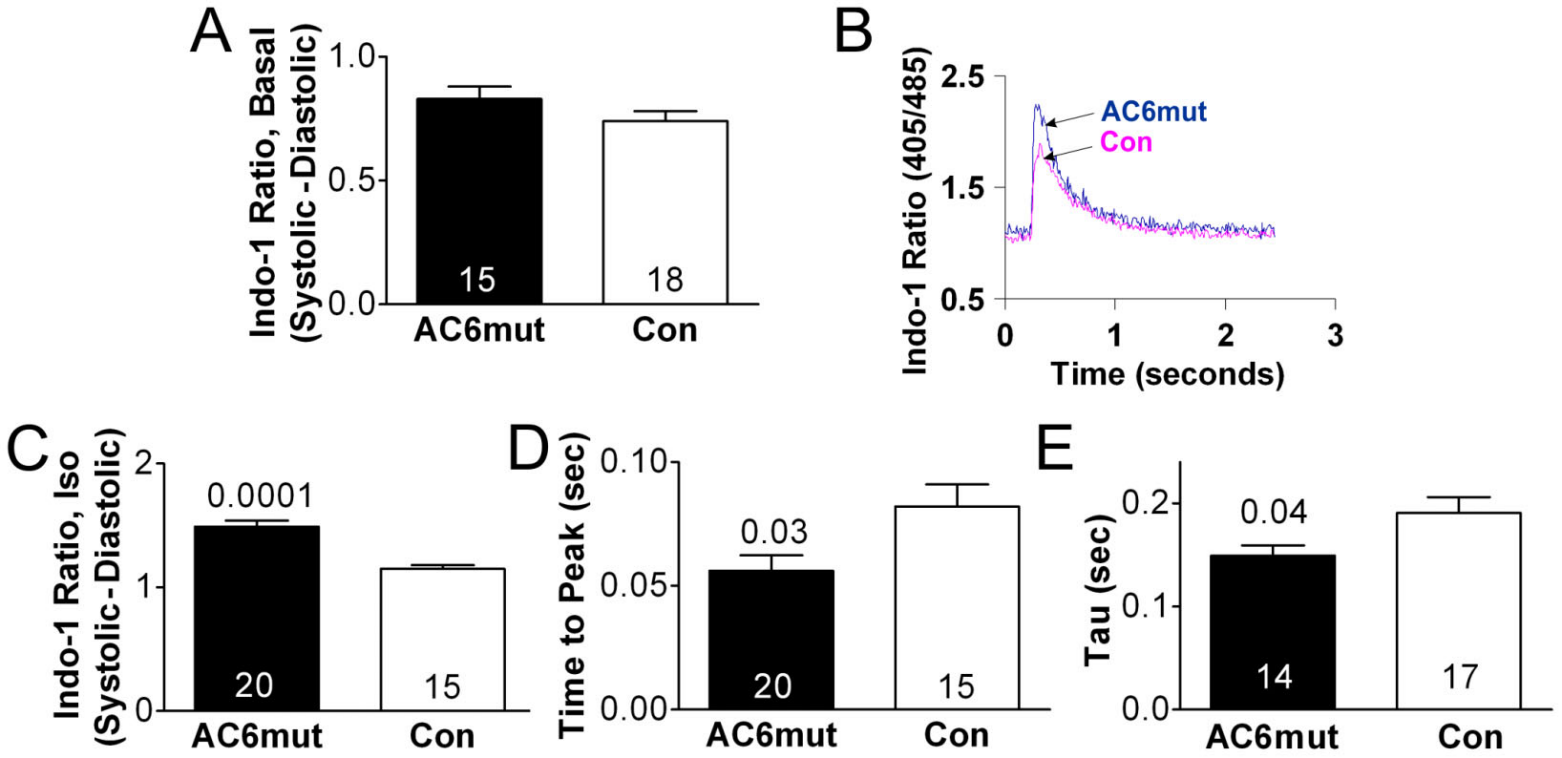
Cytosolic Ca^2+^ transients in isolated cardiac myocytes from AC6mut and control mice. **A**. Basal Ca^2+^ released (systolic-diastolic Ca^2+^) showed no group difference. **B**. Representative Indo-1 Ca^2+^ transient recordings in cardiac myocytes stimulated with isoproterenol (Iso; 10 µM) were higher in cardiac myocytes from AC6mut mice. Summary data are displayed in Panel C. **C**. Ca^2+^ released in the presence of isoproterenol was increased in cardiac myocytes from AC6mut mice. **D**. Time-to-peak Ca^2+^ transient in the presence of isoproterenol was decreased in cardiac myocytes from AC6mut mice. **E**. Time to 50% relaxation (tau) in the presence of isoproterenol was decreased in cardiac myocytes from AC6mut mice. Experiments were repeated four times. Bars denote mean +SE; numbers in bars indicate number of cardiac myocytes; numbers above bars indicate p values from Student's t-test (unpaired, 2-tailed).

To determine whether increased SR Ca^2+^ uptake associated with AC6mut expression would affect cytosolic [Ca^2+^]i, cardiac myocyte real-time [Ca^2+^]i was assessed using the ratiometric dye Indo-1. Basal Ca^2+^ release during contraction was unchanged (**Fig. 5A**). However, AC6mut expression was associated with increased peak systolic *Ca^2+^* transient amplitude after isoproterenol stimulation (p = 0.0001, **Figs. 5B and 5C**), and time to peak amplitude was decreased (p = 0.03, **Fig. 5D**). In addition, time to 50% relaxation (tau) was decreased (p = 0.04) in cardiac myocytes from AC6mut mice (**Fig. 5E**). Thus, SERCA2a activity, expression of PLB and S100A1, and isoproterenol-stimulated Ca^2+^ transients all were altered by AC6mut expression in a manner that would favorably influence LV function.

## Discussion

The most surprising and important finding of this study is that cardiac-directed expression of a mutant AC6 molecule that markedly impairs βAR-stimulated cAMP production is associated with preserved LV function in response to isoproterenol stimulation. This was confirmed by echocardiography and studies of contractile function in isolated perfused hearts. Marked diminution of cardiac cAMP generation in other settings is associated with proportional reductions in LV contractile function. For example, most models of heart failure, where cAMP impairment typically is 50% reduced, there is a similar reduction in LV dP/dt and in βAR-responsiveness [10],[11],[12],[13],[14]. Furthermore, deletion of AC6, which is associated with a 60% reduction in cAMP generating capacity, was also associated with a similar reduction in LV contractile function [5]. What then explains preservation of isoproterenol-stimulated LV contractile function?

The proximate mechanisms for preserved LV function despite markedly impaired cAMP generation in the AC6mut line were favorable changes on Ca^2+^ handling. We previously reported that cardiac-directed expression of AC6 increased function of the failing heart, but because of pronounced effects of AC6 on βAR signaling, it was impossible to determine the degree to which these beneficial effects reflected augmented βAR signaling per se vs Ca^2+^ handling [10],[11]. Supporting the link of AC6 to Ca^2+^ handling is the observation that AC6 deletion has striking adverse effects on Ca^2+^ handling [5], but since cAMP-generating capacity was reduced following AC6 deletion, the independent effects of AC6 on Ca^2+^ handling were difficult to ascertain. What is new in the present study, however, is the demonstration in TG mice that an AC6 mutant molecule appears to mimic the parent molecule's favorable effects on Ca^2+^ handling, thereby preserving LV function even whilst cAMP generating capacity is markedly diminished. It appears that the effects of AC6 on Ca^2+^ handling does not require cAMP generation, and must therefore occur through alternative mechanisms.

We found that AC6mut expression is associated with increased SR Ca^2+^ uptake in LV homogenates and increased Ca^2+^ transients with reduced time of relaxation in intact cardiac myocytes. Associated with these physiologically favorable effects of AC6mut expression was reduced PLB expression, a Ca^2+^ regulator that inhibits SERCA2a activity. Reduced PLB content or increased PLB phosphorylation at Ser16 is associated with reduction of its inhibitory effects, which increases SERCA2a activity [20],[21],[22]. We previously found that PLB expression is reduced in cultured cardiac myocytes expressing AC6 or AC6mut [4], but the current study is the first to demonstrate that this effect is also seen in vivo (**Fig. 4B**). Increases in the degree of isoproterenol-stimulated phosphorylation of RyR2, PLB, and to a lesser extent, TnI (**Fig. 2C**) in cardiac myocytes isolated from AC6mut mice would be predicted also to increase LV contractile function.

AC6mut expression was associated with increased expression and nuclear translocation of CREM-1 (**Figs. 3B and 3E**), a transcriptional suppressor in the CREB/ATF family [23]. We previously identified that, in the setting of AC6 expression, the PLB promoter was negatively regulated by increased ATF3 in neonatal rat cardiac myocytes through the CRE site in the PLB promoter [2]. In the present study we did not see increased ATF3 expression associated with AC6mut expression. However, both ATF3 and CREM-1 recognize the same CRE sites, so it is plausible that the AC6mut-related increased CREM-1 may be mechanistically important in reduced PLB expression. This will require additional study.

AC6mut expression was associated with an unanticipated increase in LV expression of the Ca^2+^ sensitizing protein, S100A1, which increases contractile function through modulation of RyR2 and SERCA2a [24]. How might AC6mut expression be linked with increased LV S100A1 expression? AC6mut expression was associated with increased phosphorylation and nuclear translocation of CREB (**Figs. 4C and 4E**), processes that are required for CREB activation. CREB is a transcriptional activator that regulates many genes through CRE site(s) in their promoters [25]. The S100A1 promoter possesses a CRE site [26], indicating that S100A1 expression could plausibly have been activated by AC6mut expression. In addition, compartmentalization of PKA and cAMP may also be factors [27],[28].

The substantial improvements in Ca^2+^ handling appear to have preserved LV function despite marked diminution in cAMP generation. The precise pathways by which increased amounts of AC6mut influence transcriptional regulation and ultimately the physiological behavior of cardiac myocytes and LV function will require additional studies. Histological studies (**Fig. 1E**) confirm that substantial amounts of transgene AC6mut are present in multiple intracellular compartments, not just in the plasma membrane. This enables AC6mut protein to interact with important intracellular proteins that influence intracellular signaling and thereby affect physiological function.

The importance of AC6 vis-à-vis Ca^2+^ handling was recently underscored by AC6 deletion [5]. In this setting, cAMP generating capacity was reduced, albeit not by as much as in the present study, but Ca^2+^ handling was markedly impaired. In the present study, we see more marked impairment of cAMP generation, but Ca^2+^ handling is increased, not decreased. This is because, unlike in AC6 deletion, the AC6 molecule, albeit one deficient in cAMP generating capacity, is present in the cytoplasm where it may influence Ca^2+^ handling.

A potential limitation of the present study is that we did not examine transgenic lines that expressed reduced amounts of AC6mut to determine if the physiological effects were proportional to level of AC6mut expression. One could argue that a 17-fold increase in AC6mut protein (vs endogenous AC6) might affect signaling in a non-specific manner. While our data cannot discount this possibility, it is important to recognize that endogenous AC6 is an exceedingly low abundance protein—approximately 100-fold less abundant, for example, than Gsα [29]. Therefore, even expressed at 17-fold higher level than endogenous AC6, it still is considerably less abundant than Gsα. Furthermore, similar increases in the catalytically active (normal) AC6 are associated with marked increases in recruitable cAMP production [30]. These observations suggest that the findings are specific.

## Conclusions

Substantial improvements in Ca^2+^ handling appear to preserve LV function despite marked diminution in cAMP generation. Immunofluorescence indicates that AC6mut is located on the nuclear envelope, providing an opportunity for AC6mut to influence transcription factor expression and function. Increased CREM-1, a transcriptional suppressor and increased phospho-CREB (**Fig. 4E**) may be involved in altered expression of PLB and S100A1 respectively. We conclude that AC6mut preserves cardiac function through increased Ca^2+^ handling and altered protein expression, despite reduced cAMP generation. These results provide insight regarding the interplay between Ca^2+^ handling and βAR signaling vis-à-vis LV function, and indicate that AC6mut may provide inotropic stimulation free from the potentially deleterious effects of increased cAMP. It will be interesting to see what effects AC6mut expression has on the failing heart. Preliminary data indicate reduced cardiac myocyte apoptosis associated with AC6mut expression in the failing heart, which is a focus of an ongoing study in our laboratory.

### Clinical perspective

So many clinical trials of positive inotropes have failed, that it is now axiomatic that agents that increase cAMP are deleterious to the failing heart. An alternative strategy is to alter myocardial Ca^2+^ handling or myofilament response to Ca^2+^ using agents that do not affect cAMP. Expression of a catalytically impaired AC6 mutant molecule — one that markedly reduces cAMP production — is associated with normal cardiac function in response to β-adrenergic receptor stimulation. The mechanism is through enhanced effects of AC6mut on Ca^2+^ handling — effects that do not require cAMP. These data are important in clinical settings for two reasons: 1) the results provide additional insight regarding the interplay between Ca^2+^ handling and βAR signaling vis-à-vis LV function; and 2) AC6mut may provide inotropic support free from the potentially deleterious effects of increased cAMP. Ongoing studies will be required to determine the effects of AC6mut gene transfer in the setting of heart failure.
